# A meta-analysis between dietary carbohydrate intake and colorectal cancer risk: evidence from 17 observational studies

**DOI:** 10.1042/BSR20160553

**Published:** 2017-04-10

**Authors:** Jian Huang, Guoqing Pan, Hongchao Jiang, Wenliang Li, Jian Dong, Hongbin Zhang, Xiang Ji, Zhu Zhu

**Affiliations:** 1 Department of Cancer Center, The First Affiliated Hospital of Kunming Medical University, Kunming, Yunnan 650032, P.R. China; 2 Department of Cancer Center, The Third Affiliated Hospital of Kunming Medical University, Kunming, Yunnan 650032, P.R. China

**Keywords:** Colorectal cancer, Colon cancer, Dietary carbohydrate intake, Meta-analysis, Recrum cancer

## Abstract

The association between dietary carbohydrate intake and colorectal cancer (CRC) risk remains controversial. We therefore conducted this meta-analysis to assess the relationship between them. A literature search from the databases of PubMed, Embase, Web of Science and Medline was performed for available articles published in English (up to September 2016). Pooled relative risk (RR) with 95% confidence interval (CI) was calculated to evaluate the association between dietary carbohydrate intake and CRC risk. The random-effect model (REM) was selected as the pooling method. Publication bias was estimated using Egger’s regression asymmetry test and funnel plot. A total of 17 articles involving 14402 CRC patients and 846004 participants were eligible with the inclusion criteria in this meta-analysis. The pooled RR with 95% CI of dietary carbohydrate intake for CRC, colon cancer and rectum cancer risk were 1.08 (95% CI =0.93–1.23, *I^2^* =68.3%, *P*_heterogeneity_<0.001), 1.09 (95% CI =0.95–1.25, *I^2^* =48.3%) and 1.17 (95% CI =0.98–1.39, *I^2^* =17.8%) respectively. When we conducted the subgroup analysis by gender, the significant association was found in men’s populations (summary RR =1.23, 95% CI =1.01–1.57), but not in the women’s populations. In the further subgroup analyses for study design and geographic locations, we did not find any association between dietary carbohydrate intake and CRC risk in the subgroup results respectively. No significant publication bias was found either by the Egger’s regression asymmetry test or by the funnel plot. This meta-analysis suggested that higher dietary carbohydrate intake may be an increased factor for CRC risk in men populations. Further studies are wanted to confirm this relationship.

## Introduction

Colorectal cancer (CRC) is the third most common cancer and the third most common cause of high mortality cancer rates in the United States [1]. Although the incidence of CRC is 18% higher in developed regions in comparison with lower income areas [2], there is a rapid increase in developing countries in the past few decades, which makes it a major public health concern worldwide. Several lines of evidence indicate that insulin resistance may play a role in the aetiology of CRC. Some risk factors for CRC including overweight and obesity, low physical activity and Type II diabetes are linked to insulin resistance [3,4].

Dietary carbohydrate intake is the main dietary component affecting an individual’s insulin secretion and glycaemic response, but the effect varies depending on the amount of carbohydrate consumed [5]. Although many studies were conducted to assess the association between dietary carbohydrate intake on CRC risk up to now, there is no comprehensive conclusion. For higher category of dietary carbohydrate intake, a prospective study by Higginbotham et al. [6] reported that it could increase the CRC risk. In contrast, another prospective study by Strayer et al. [7] showed that higher category of dietary carbohydrate intake was a protected factor on CRC risk. To our attention, many of the observational studies [8–11] did not find the association between them. Thus, we performed a meta-analysis to (i) assess the effect of dietary carbohydrate intake on CRC risk, (ii) explore the potential heterogeneity among studies and (iii) assess the potential publication bias.

## Materials and methods

### Search strategy

A search was conducted for available articles that were published in English (up to September 2016) from the following databases: (i) PubMed, (ii) Web of Science, (iii) Embase and (iv) Medline. Search terms included ‘carbohydrate’ OR ‘nutrition’ OR ‘dietary’ AND ‘colorectal cancer’ OR ‘colon cancer’ OR ‘rectum cancer’ without any restriction. Moreover, we reviewed the reference lists of the studies included to identify additional studies not captured by our database searches.

### Inclusion criteria

The inclusion criteria were as follows: (i) observational study published as the original study to assess the association between dietary carbohydrate intake and CRC risk; (ii) the exposure of interest was dietary carbohydrate intake; (iii) the outcome of interest was CRC; (iv) relative risk (RR) or odds ratio (OR) with corresponding 95% confidence interval (CI) (or data to calculate them) were available; (v) the complete and recent study was chosen if a study had been published more than once.

All included studies were independently reviewed by two investigators carefully to determine and identify whether an individual study was eligible for inclusion in this meta-analysis. Articles for which it was unclear whether or not eligible for the inclusion criteria were discussed at consensus meetings including both investigators.

### Data extraction

Two investigators who reached a consensus on all of the items independently extracted the data. The following information was extracted from each study: the first author’s name, publication year, mean age or age range, country, gender, study design, disease type, number of cases and participants, variables adjusted for the analysis and RR (we presented all results with RR for simplicity) with their 95% CI.

### Statistical analysis

When available, we extracted the RR adjusted with the most potential confounders in the original articles. Pooled RR was calculated as the inverse variance-weighted mean of the logarithm of multivariate adjusted RR with their 95% CI to evaluate the association between dietary carbohydrate intake and CRC risk [12]. For articles that reported results separately for women and men or different age groups, CRC, colon cancer and rectum cancer, but not combined, we extracted the data as a separate study. Heterogeneity among studies was assessed with the *I^2^* [13] and *I^2^* values of 75, 50, 25 and 0% represent high, moderate, low and no heterogeneity [14] respectively. The random-effect model (REM) was selected as the pooling method. Meta-regression was conducted to evaluate the potentially important covariates that might have substantial impacts on between-study heterogeneity [15]. Publication bias was assessed with the funnel plot and Egger’s test [16]. STATA version 12.0 was used to perform the statistical analyses. All reported probabilities (*P* values) were two sided at the level of 0.05.

## Results

### Search results and study characteristics

**Figure 1:**
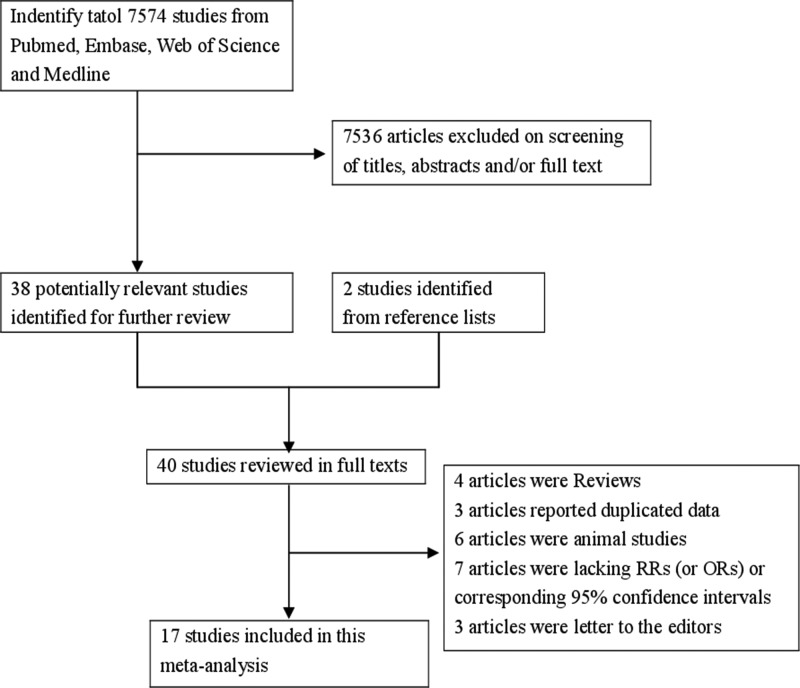
The flow diagram of screened, excluded and analysed publications. The flow diagram of screened, excluded and analysed publications.

In our search strategy, 7574 articles from the databases of PubMed, Embase, Web of Science and Medline were selected. After reviewing the title/abstract, 38 articles were reviewed in full, 2 studies identified from reference list, 23 of which were subsequently excluded due to the following reasons: four articles were reviews, three articles reported duplicated data, six articles were animal studies, seven articles were lacking RR (or OR) or corresponding 95% CIs and three articles were letters to the editors. Ultimately, 17 articles [6–11,17–27] involving 14402 CRC patients and 846004 participants were eligible for the inclusion criteria. Among these studies, eight were in case-control design and nine in prospective design. Twelve studies were carried out in America, two in Europe and three in Asia. The detailed step of the literature search is shown in Figure 1. The characteristics of included studies are listed in Table 1.

**Table 1:** Characteristics of studies on dietary carbohydrate intake with the risk of CRC

Study (year)	Country	Study design	Participants (cases)	Age (years)	RR (95% CI) for highest compared with lowest category	Adjustment for covariates
Borugian, M.J. (2002)	North America	Case-control	1665 (473)	67.6 ± 12.2	Colorectal	Adjusted for age, education, family history, QI, years in North America, total kilocalories consumed, and intake of fat, calcium and fibre
					1.7 (1.1–2.7) for men	
					2.7 (1.5–4.8) for women	
					Colon	
					1.6 (0.9–3.1) for men	
					1.7 (0.7–4.0) for women	
					Rectum	
					2.4 (1.2–4.8) for men	
					2.7 (1.2–6.2) for women	
De Stefani, E. (2012)	Uruguay	Case-control	1973 (611)	NA	Colorectal	Adjusted for age, sex, residence, urban/rural status, education, family history of colon cancer among first-degree relatives, BMI, smoking intensity, smoking duration in years, alcohol drinking and total energy intake
					1.28 (0.96–1.70)	
					Colon	
					1.46 (1.02–2.09)	
					Rectum	
					1.10 (0.75–1.61)	
Ghadirian, P. (1997)	Canada	Case-control	1070 (402)	35–79	Colon	Adjusted for gender, age, marital status, history of colon carcinoma in first-degree relatives and total energy intake
					0.86 (0.59–1.26)	
Higginbotham, S. (2004)	American	Cohort	38451 (174)	53.9	Colorectal	Adjusted for age, BMI, history of oral contraceptive use, post-menopausal hormone use, family history of CRC, smoking (never, past, current), alcohol use, physical activity, non-steroidal anti-inflammatory use (never/rarely, >1 time per week), total energy intake, energy adjusted total fibre (g), energy adjusted total fat (g), energy-adjusted folate (μg), energy-adjusted calcium (mg) and energy-adjusted vitamin D (mg)
					2.41 (1.10–5.27) for women	
Howarth, N.C. (2008)	American	Cohort	191004 (2086)	45–75	Colorectal	Adjusted for age, ethnicity and time since cohort entry; restricted to subjects with no missing values for family history of CRC, history of colorectal polyp, pack years of cigarette smoking, BMI, hours of vigorous activity, non-steroidal anti-inflammatory drug use, multivitamin use and replacement, hormone use (women only)
					1.09 (0.84–1.40) for men	
					0.71 (0.53–0.95) for women	
					Colon	
					1.10 (0.81–1.49) for men	
					0.69 (0.50–0.96) for women	
					Rectum	
					0.98 (0.60–1.59) for men	
					0.78 (0.42–1.44) for women	
Iscovich, J.M. (1992)	Argentina	Case-control	330 (110)	NA	Colon	Adjusted for fibre at 19.02 g per day, other sources of energy intake
					4.46 (1.45–13.71)	
Kabat, G.C. (2008)	American	Cohort	158800 (1476)	50–79	Colorectal	Adjusted for age (continuous), education, cigarettes smoked per day, BMI (continuous), height (continuous), hormone replacement therapy (ever, never), history of diabetes (no, yes), family history of CRC in a first-degree relative (yes, no), total metabolic equivalent hours per week from physical activity (continuous), Observational Study participant (yes, no) and intake of total fibre, energy (kcal) and dietary calcium
					0.89 (0.64–1.25) for women	
					Colon	
					0.78 (0.49–1.25) for women	
					Rectum	
					1.33 (0.62–2.85) for women	
Larsson, S.C. (2007)	Sweden	Cohort	61433 (870)	40–76	Colorectal	Adjusted for age in months and date of enrollment and included the following: education, BMI (weight (kg)/height (m^2^); <23, 23 to <25, 25 to <30 or 30), total energy intake (continuous) and quartiles of intake of alcohol, cereal fibre, folate, calcium, magnesium and red meat
					1.10 (0.85–1.44) for women	
					Colon	
					1.14 (0.83–1.57) for women	
					Rectum	
					0.94 (0.59–1.50) for women	
Li, H.L. (2011)	China	Cohort	73061 (475)	40–70	Colorectal	Adjusted for age, education, income, BMI, physical activity, family history of CRC, total energy intake and hormone replacement therapy use by using a Cox model with age as the time scale and stratified by birth year
					0.87 (0.66–1.15) for women	
					Colon	
					0.79 (0.55–1.12) for women	
					Rectum	
					1.02 (0.66–1.59) for women	
Michaud, D.S. (2005)	American	Cohort	173229 (1779)	30–75	Colorectal	Adjusted for age, family history of colon cancer, prior endoscopy screening, aspirin use, height, BMI, pack years of smoking before the age of 30, physical activity and intake of cereal fibre, alcohol, calcium, folate, processed meat and beef, pork or lamb as the main dish
					1.27 (0.93–1.72) for men	
					0.87 (0.68–1.11) for women	
					Colon	
					1.21 (0.85–1.71) for men	
					0.86 (0.65–1.13) for women	
					Rectum	
					1.45 (0.73–2.38) for men	
					0.91 (0.53–1.55) for women	
Sieri, S. (2015)	Italy	Cohort	44225 (421)	NA	Colorectal	Adjusted for education, smoking status, BMI, alcohol intake, calcium intake, folate intake, fibre intake, saturated fat intake, non-alcohol energy and physical activity
					1.51 (0.97–2.34)	
					Colon	
					1.20 (0.81–1.79)	
					Rectum	
					1.14 (0.47–2.78)	
Slattery, M.L. (1997)	American	Case-control	4393 (1983)	30–79	Colon	Adjusted for age, BMI, family history of first-degree relative with CRC, use of aspirin and/or non-steroidal anti-inflammatory drugs, physical activity and dietary intake of fibre, cholesterol, and calcium
					1.47 (0.98–2.22) for men	
					1.27 (0.82–1.97) for women	
Strayer, L. (2007)	American	Cohort	45561 (490)	61.9	Colorectal	Adjusted for age, dietary calories, NSAIDS use, smoking, menopausal female hormone use, screened for CRC, BMI and fibre intake
					0.70 (0.50–0.97) for women	
Sun, Z. (2012)	Canada	Case-control	4241 (1760)	20–74	Colorectal	Adjusted for total energy intake. Other potential confounders included age, sex, BMI, physical activity, family history of CRC, polyps, diabetes, reported colon screening procedure, cigarette smoking, alcohol drinking, education attainment, household income, marital status, regular use of NSAID, regular use of multivitamin supplements, regular use of folate supplement, regular use of calcium supplement, reported HRT (females only), province of residence, and intake of fruits, vegetables, and red meat. Variables were included in the final model based on a ≥10% alternation in the parameter coefficient of interest
					0.81 (0.63–1.00)	
Tayyem, R.F. (2015)	Jordan	Case-control	417 (169)	53.8 ± 12.2	Colorectal	Adjusted for total energy intake normality of the distributions of dietary intake variables was assessed by the Shapiro–Wilk test. Non-normally distributed variables were log transformed. Other potential confounders included age, gender, BMI, physical activity (METs/week), family history (beyond the second degree) of CRC, education attainment, household income, marital status and tobacco use
					1.41 (0.68–2.99)	
Terry, P.D. (2003)	American	Cohort	49124 (616)	40–59	Colorectal	Adjusted for age, intake of energy, study centre, treatment allocation, BMI (quartiles), cigarette smoking, educational level, physical activity, oral contraceptive use, hormone replacement therapy, parity (quintiles) and quartiles of alcohol, red meat and folic acid
					1.01 (0.68–1.51) for women	
					Colon	
					1.04 (0.63–1.72) for women	
					Rectum	
					0.98 (0.49–1.97) for women	
Wakai, K. (2006)	Japan	Case-control	3042 (507)	20–79	Colon	Adjusted for age, sex, year of first visit, season of first visit to the hospital, reason for the visit, family history of CRC, BMI, exercise, alcohol drinking, smoking, multivitamin use, and energy intake
					1.16 (0.76–1.79)	
					Rectum	
					1.54 (0.96–2.47)	

BMI, body mass index.

### Overall analysis and subgroup analyses

We pooled the overall meta-analyses to assess the association between dietary carbohydrate intake and CRC risk. Subgroups analyses were also conducted by study type, geographic locations, sex and disease type. The detailed summary risk estimates are summarized in Table 2.

**Table 2:** Combined results of dietary carbohydrate intake with the risk of CRC

Subgroups	Number of cases	Number of studies	RR (95% CI)	*I^2^* (%)	*P*_heterogeneity_
All studies	11400	16	1.08 (0.93–1.23)	68.3	<0.001
Disease type					
Colon	9235	17	1.09 (0.95–1.25)	48.3	0.014
Rectum	3272	13	1.17 (0.98–1.39)	17.8	0.264
Study design					
Cohort	8387	11	0.99 (0.85–1.15)	57.4	0.009
Case-control	3013	5	1.40 (0.93–2.09)	80.8	<0.001
Sex					
Both	2961	4	1.16 (0.83–1.62)	69.8	0.019
Men	2133	3	1.23 (1.01–1.57)	30.9	0.235
Women	6306	9	0.99 (0.81–1.23)	70.3	0.001
Geographic locations					
America	9465	12	1.08 (0.89–1.30)	73.6	<0.001
Asia	644	2	0.98 (0.65–1.46)	30.2	0.231
Europe	1291	2	1.23 (0.91–1.64)	31.7	0.226

**Figure 2:**
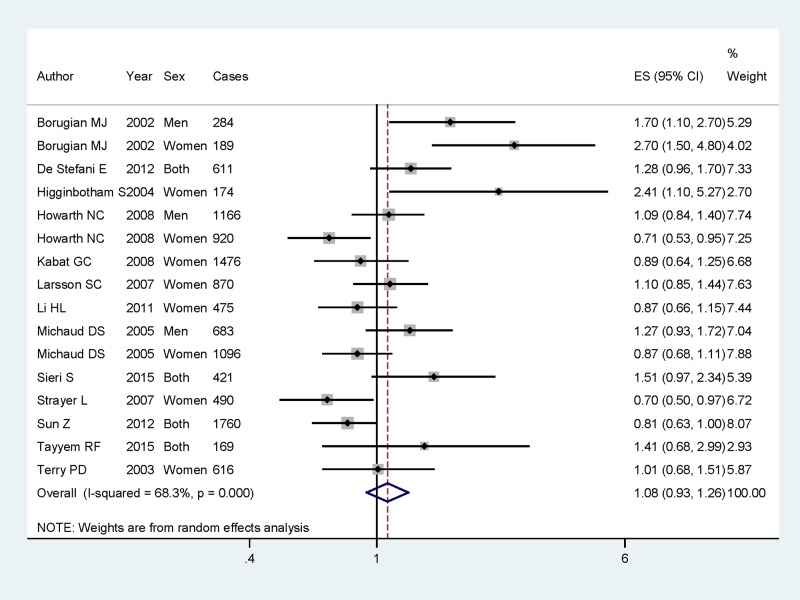
The forest plot between highest compared with lowest categories of dietary carbohydrate intake and CRC risk. The forest plot between highest compared with lowest categories of dietary carbohydrate intake and CRC risk.

**Figure 3:**
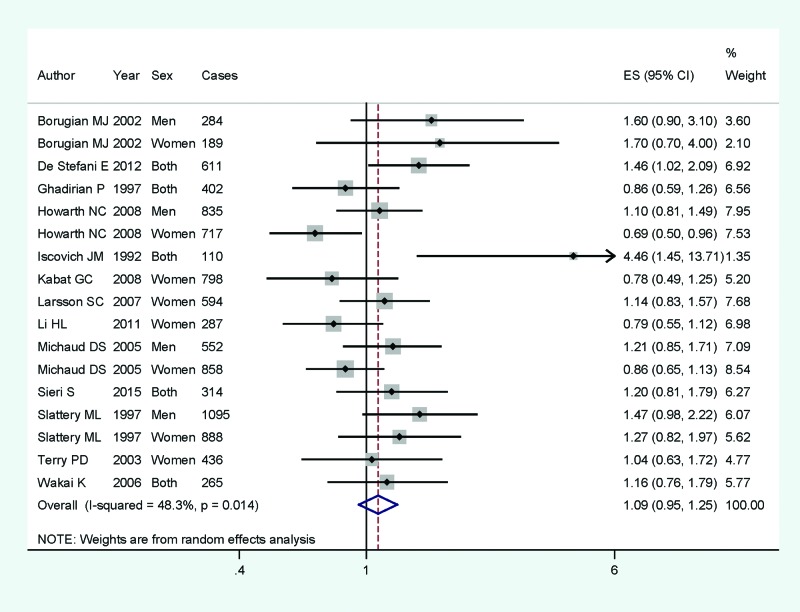
The forest plot between highest compared with lowest categories of dietary carbohydrate intake and colon cancer risk. The forest plot between highest compared with lowest categories of dietary carbohydrate intake and colon cancer risk.

**Figure 4:**
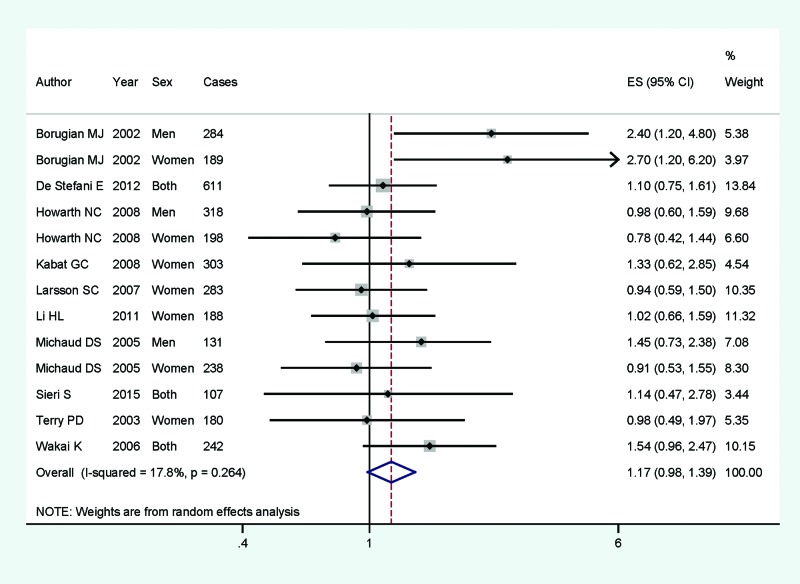
The forest plot between highest compared with lowest categories of dietary carbohydrate intake and rectum cancer risk. The forest plot between highest compared with lowest categories of dietary carbohydrate intake and rectum cancer risk.

Of the 17 articles included, 16 studies involving 11400 CRC cases and 840171 participants were reported the association between dietary carbohydrate intake and CRC risk; 17 studies with 9235 cases were to assess the association on colon cancer; and 13 studies with 3272 cases were to assess the association on rectum cancer. Pooled results suggested that higher category of dietary carbohydrate intake had no significant association for CRC risk (summary RR =1.08, 95% CI =0.93–1.23, *I^2^* =68.3%, *P*_heterogeneity_<0.001) (Figure 2). The associations were also not significant in colon cancer (summary RR =1.09, 95% CI =0.95–1.25, *I^2^* =48.3%) (Figure 3) or rectum cancer (summary RR =1.17, 95% CI =0.98–1.39, *I^2^* =17.8%) (Figure 4) for higher category of dietary carbohydrate intake. In subgroup analyses for study design, the association was not significant either in the case-control studies (summary RR =1.40, 95% CI =0.93–2.09] or in the cohort studies (summary RR =0.99, 95% CI =0.85–1.15]. In stratified analysis by geographic locations, higher dietary carbohydrate intake had no significant association on CRC risk among American populations (summary RR =1.08, 95% CI =0.89–1.30), European populations (summary RR =1.23, 95% CI =0.91–1.64] or Asian population (summary RR =0.98, 95% CI =0.65–1.46). When we conducted the subgroup analysis by gender, the significant association was found in men populations (summary RR =1.23, 95% CI =1.01–1.57), but not in the women populations.

### Sources of heterogeneity

As shown in Figure 2, strong evidence of between-study heterogeneity (*I^2^* =68.3%, *P*_heterogeneity_<0.001) was demonstrated for dietary carbohydrate intake and CRC risk. Therefore, univariate meta-regression with covariates was conducted. However, the results showed that no covariates including publication year, gender, study type, disease type and study region had a significant impact on heterogeneity. Considering three included studies [6,17] showing a high risk ratio, in contrast with most of the other studies, we then pooled the whole result with excluding these high risk ratio studies. The between-study heterogeneity was reduced to 28.3%, *P*_heterogeneity_=0.231. The result for highest category of dietary carbohydrate intake for CRC risk was consistent with the whole result (summary RR =0.98, 95% CI =0.86–1.11).

### Influence analysis

Influence analysis showed that no individual study was found to have excessive influence on the pooled estimate for dietary carbohydrate intake and CRC risk.

### Publication bias

**Figure 5:**
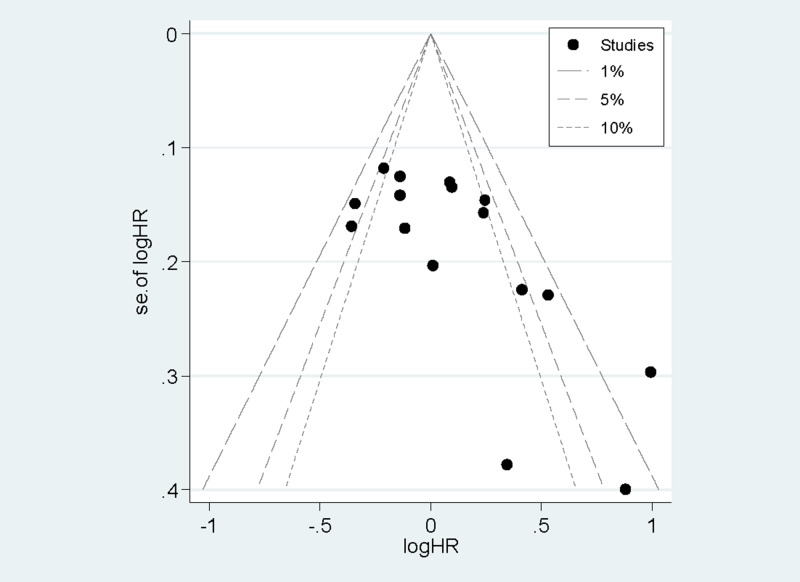
The funnel plot of the association between dietary carbohydrate intake and CRC risk. The funnel plot of the association between dietary carbohydrate intake and CRC risk.

Egger’s test (*P*=0.14) and the funnel plot (Figure 5) demonstrated that no evidence of significant publication bias was found for dietary carbohydrate intake and CRC risk.

## Discussion

In recent years, studies have been conducted to assess the association between dietary carbohydrate intake and CRC risk. However, the results remained conflicting. Hence, we conducted this meta-analysis. The findings from the results indicate that no significant associations were found between higher dietary carbohydrate intake and CRC risk in overall results or in subgroup analyses by study design, disease type and geographic locations respectively. However, we found that it increased the risk of CRC with higher dietary carbohydrate intake among men’s populations, but not women. In the present meta-analysis, only three studies were included to assess this relationship for men populations. Therefore, the result is not very stable during small studies. Further studies are wanted to confirm this problem.

Heterogeneity among studies is common in meta-analysis [28], and it is necessary to explore the potential causes of heterogeneity. As it is shown, strong evidence of between-study heterogeneity was demonstrated in our meta-analysis. So we conducted meta-regression to explore the potential sources of heterogeneity among studies. However, the results showed that no covariates including publication year, gender, study type, disease type and study region had a significant impact on heterogeneity. We then conducted the subgroup analyses to explore the heterogeneity, but the heterogeneity was high in some subgroup analyses. Considering three included studies showing a high risk ratio, in contrast with most of the other studies, we then pooled the whole result with excluding these high risk ratio studies. The pooled RR with 95% CI of dietary carbohydrate intake for CRC risk was 0.98 (95% CI =0.86–1.11). And the between-study heterogeneity was 28.3%, *P*_heterogeneity_=0.231). Therefore, these three studies were the potential causes of heterogeneity.

As a meta-analysis, our study has several strengths. Firstly, large number of participants included reduced sampling error to a great extent to reach more reasonable conclusions. Secondly, most of all studies on dietary carbohydrate intake and CRC risk had adjusted for major risk factors, such as family history of colon cancer among first-degree relatives, smoking intensity, alcohol drinking and total energy intake, suggesting that the results were more reliable. Thirdly, most of the studies were prospective, allowing a much greater possibility of reaching reasonable conclusions. As prospective studies do not suffer from recall bias and are anticipated to be less likely to have selection bias relative to case-control studies. Finally, no significant publication bias was found, indicating that our results are stable.

However, this meta-analysis also has some limitations. Firstly, as we all know, observational studies are susceptible to potential bias inherent in the original studies, especially for case-control studies in the meta-analysis. There is some case-control studies included in this meta-analysis. Overstated association could be expected from the case-control studies because of recall or selection bias, and early symptoms in patients may have resulted with a change in dietary habits, although we found no differences in risks between cohort studies and case-control studies. Secondly, although we extracted the multivariable-adjusted RRs, the extent to which they were adjusted in the original studies varied. Thirdly, most of the included studies came from America, however, no significant association was found. There are only two studies that came from Asia and two studies came from Europe, which are difficult to obtain reliable conclusions with such a small sample size for Asian and European populations. Therefore, more studies conducted from Asia and Europe are wanted to assess this relationship. Finally, despite the correction for potential confounders, the RR ratio remains an association. Considering the variability of results in the included studies, this would influence the final result of the study. Therefore, further studies with more cases and participants are wanted to confirm the association.

In summary, results from this meta-analysis suggested that higher dietary carbohydrate intake may be an increased risk factor for CRC risk in men populations. Further studies are wanted to confirm this relationship.

## Abbreviations

CI: confidence interval
CRC: colorectal cancer
HRT: hormone replacement therapy
MET: metabolic equivalents
NSAIDS: non-steroidal anti-inflammatory drugs
OR: odds ratio
QI: Quetelet's index
RR: relative risk
